# Establishment and characterization of *in vivo* orthotopic bioluminescent xenograft models from human osteosarcoma cell lines in Swiss nude and NSG mice

**DOI:** 10.1002/cam4.1346

**Published:** 2018-02-23

**Authors:** Maria Eugenia Marques da Costa, Estelle Daudigeos‐Dubus, Anne Gomez‐Brouchet, Olivia Bawa, Valerie Rouffiac, Massimo Serra, Katia Scotlandi, Conceição Santos, Birgit Geoerger, Nathalie Gaspar

**Affiliations:** ^1^ Vectorology and Anticancer Therapies UMR 8203, CNRS University of Paris‐Sud Gustave Roussy, Université Paris‐Saclay Villejuif France; ^2^ CESAM & Department of Biology University of Aveiro Aveiro Portugal; ^3^ Department of Pathology IUCT‐Oncopole CHU of Toulouse and University of Toulouse Toulouse France; ^4^ Pharmacology and Structural Biology Institut CNRS UMR5089 Toulouse France; ^5^ Plateforme HistoCytoPathologie UMS AMMICa, Gustave Roussy Villejuif France; ^6^ Imaging and Cytometry Platform UMS 3655& US23 Gustave Roussy, Paris‐Saclay University Villejuif France; ^7^ Laboratory of Experimental Oncology Orthopaedic Rizzoli Institute Bologna Italy; ^8^ Department of Biology Faculty of Sciences University of Porto Porto Portugal; ^9^ Department of Oncologie for child and adolescent Gustave Roussy Villejuif France

**Keywords:** Bioluminescence, cell‐derived xenograft, human osteosarcoma, *in vivo* orthotopic

## Abstract

Osteosarcoma is one of the most common primary bone tumors in childhood and adolescence. Metastases occurrence at diagnosis or during disease evolution is the main therapeutic challenge. New drug evaluation to improve patient survival requires the development of various preclinical models mimicking at best the complexity of the disease and its metastatic potential. We describe here the development and characteristics of two orthotopic bioluminescent (Luc/mKate2) cell‐derived xenograft (CDX) models, Saos‐2‐B‐Luc/mKate2‐CDX and HOS‐Luc/mKate2‐CDX, in different immune (nude and NSG mouse strains) and bone (intratibial and paratibial with periosteum activation) contexts. IVIS SpectrumCT system allowed both longitudinal computed tomography (CT) and bioluminescence real‐time follow‐up of primary tumor growth and metastatic spread, which was confirmed by histology. The murine immune context influenced tumor engraftment, primary tumor growth, and metastatic spread to lungs, bone, and spleen (an unusual localization in humans). Engraftment in NSG mice was found superior to that found in nude mice and intratibial bone environment more favorable to engraftment compared to paratibial injection. The genetic background of the two CDX models also led to distinct primary tumor behavior observed on CT scan. Saos‐2‐B‐Luc/mKate2‐CDX showed osteocondensed, HOS‐Luc/mKate2‐CDX osteolytic morphology. Bioluminescence defined a faster growth of the primary tumor and metastases in Saos‐2‐B‐Luc/mKate2‐CDX than in HOS‐Luc/mKate2‐CDX. The early detection of primary tumor growth and metastatic spread by bioluminescence allows an improved exploration of osteosarcoma disease at tumor progression, and metastatic spread, as well as the evaluations of anticancer treatments. Our orthotopic models with metastatic spread bring complementary information to other types of existing osteosarcoma models.

## Background

Osteosarcoma is a rare but the most frequent primary malignant bone tumor with a peak incidence in adolescence and young adulthood 1. The survival of patients with osteosarcoma has not improved in the last 30 years since the introduction of chemotherapy in the “70s–80s” 1, 2, 3. The development of metastasis, mainly lung metastases, remains the main cause of treatment failure 4. The main prognostic factors of relapse are the metastatic status at diagnosis and the histological response to neoadjuvant chemotherapy (surrogate marker of osteosarcoma chemosensitivity) 5, 6. Several aspects might have participated in this disappointing situation, the insufficient understanding of osteosarcoma oncogenesis, the non‐optimal phase II clinical trial designs 7, and the unsatisfactory low number of preclinical osteosarcoma models.

Due to the complex osteosarcoma genetic background and the importance of bone and immune microenvironment in this tumor type 8, 9, 10, multiple osteosarcoma models representative of the human disease in different *in vitro* and *in vivo* contexts are needed to get more insight into different processes involving osteosarcoma initiation, progression especially metastatic and treatment sensitivity. The EuroBoNeT (European Network of Excellence on bone tumors) consortium has characterized 19 osteosarcoma cell lines 9, 11, 12 and described their tumorigenic capacities under simplified conditions (subcutaneous and intramuscular/paratibial xenograft conditions) to identify technically practical models 9. Although covering a large panel of osteosarcoma genetic abnormalities, these mice models might not be fully clinically relevant because osteosarcoma cells are not spontaneously arisen and do not grow in the proper site. It can be hypothesized that *in vivo* models in an orthotopic setting might reveal different tumor behavior: primary tumor growth, metastatic potential, and response to treatment 13, 14, 15, by better mimicking the initial bone site of the disease in patients. The major difficulty in using these preclinical orthotopic bone models is the measurement of the disease burden in a nonaccessible site, which requires the use of noninvasive techniques such as radiography 16, computed tomography (CT), magnetic resonance imaging (MRI), or bioluminescence 13, 14.

In this work, we used bioluminescence (cell lines transduced with luciferase) and CT imaging to facilitate *in vivo* follow‐up of primary tumor growth, changes in bone microarchitecture, and metastatic development. Therefore, we developed and characterized distinct orthotopic cell‐derived xenograft (CDX) human osteosarcoma models in mice with different immune backgrounds with metastatic potential.

## Methods

### Cell culture

A panel of seven human osteosarcoma cell lines (HOS, 143B, U2OS, MG‐63, Saos‐2, Saos‐2‐B, and IOR/OS18) mycoplasma free were used. The 143B cell line was purchased from the American Type Culture Collection. All other osteosarcoma cell lines were kindly provided within the scope of the European Consortium Innovative Therapies for Children with Cancer (ITCC). Testing Saos‐2 issued from two different culture flasks, we observed two slightly different CGH profile. We continued the experiments with both and named the second one Saos‐2‐B.

The cell lines were cultured, using early passages in Dulbecco's modified Eagle medium (DMEM, GIBCO/Invitrogen, Saint Aubin, France) supplemented with 10% (v/v) fetal bovine serum (FBS, GIBCO/Invitrogen) at 37°C in a humidified atmosphere (5% CO_2_ and 95% air). Mycoplasma test was performed each month by PCR.

### Transfection and cell transduction with Luc/mkate2 (transgene) *in vitro*

Procedures were performed in sterile and safe conditions. The procedures using genetically modified organisms (GMO) were approved by the Ministry of Higher Education and Research and performed under the conditions established according to Decree no 2011–1177. Lentiviral particles were produced by transfecting HEK 293T cells 24 h after plating, with transfection solution containing jetPRIME Transfection Reagent kit (Polyplus transfection, Illkirch, France), envelop plasmids—29.4 μg of VSVG (pMD2G) and 54.6 μg of GAGPOL (psPax2) and 48 μg of plasmid Plvx‐CAG‐luc‐2A‐mKate2 that contains the gene of interest. Plasmids were provided by David Castel from UMR8203 Research Unit, at Gustave Roussy 17. The supernatant containing the virus was collected 48 h later and centrifuged for 5 min at 2376 g and 4°C, the pellet was discarded, and the supernatant was centrifuged at 49782 g and 4°C for 70 min. The pellet was resuspended in PBS, incubated under agitation for 1 h at 4°C, centrifuged 1 min at 5000 rpm and 4°C, and aliquoted at −80°C.

For virus titration, serial dilutions of supernatants had been tested on HCT116 cells, which were then analyzed for mkate2 detection by cytometry (BD Biosciences, Le‐Pont‐De‐Claix, France), 4/5 days postinfection.

All seven cell lines were plated at 1 × 10^5^ cells per well in a 6‐well plate and infected with viral supernatant with a high MOI. After cells reached confluency, a selection of the cells marked with Luc/mKate2 was performed by flow cytometry using FACSDiva version 6.1.3. software (BD Biosciences). The cells expressing the transgene were amplified for further use.

Expression and activity were measured by bioluminescence using IVIS SpectrumCT system (Perkin Elmer, Courtaboeuf, France).

### *In vivo* bioluminescent CDX orthotopic models

Animal experiments were approved by the CEEA26, CEEA PdL No 6 Ethics Committee, and the Ministry of Agriculture (approval number: APAFIS#1648‐2015090713516480) and performed under the conditions established by the European Community (Directive 2010/63/UE).

We have established osteosarcoma orthotopic models derived from two human cell lines, using two different 7‐week‐old immunodeficient mouse strains and two different types of bone injection conditions.

#### Osteosarcoma cell lines used for CDX

Two cell lines were used for *in vivo* CDX establishment, Saos‐2‐B‐luc/mKate2 and HOS‐luc/mKate2. The nonbioluminescent human Saos‐2‐B osteosarcoma cell line was established from a primary osteosarcoma of an 11‐year‐old Caucasian female patient. In Saos‐2‐B cell line, *TP53* (del^2^ > EX4‐EX8) gene is deleted, *Rb1* mutated, and *CDKN2A* normal 9, 11, 18. Nonbioluminescent human HOS osteosarcoma cell line was established from a primary tumor of a 13‐year‐old female patient (*TP53* mutation p.Arg156Pro and *CDKN2A* homozygous deletion) 11.

#### Immunodeficient mouse strains

Swiss nude and NSG mouse strains were purchased at Gustave Roussy (Villejuif, France). They were born and bred at the animal facilities at Gustave Roussy and maintained under controlled conditions. NSG mouse strains are deficient in B and T lymphocytes and with low NK cell activity 19, minimizing the chance of xenograft rejection, while nude mouse strains have T‐cell depletion, but with age an increase in NK cells and αβTCR lymphocytes, maturation is observed. Innate immunity of the nude mice is less compromised than in the NSG strain 19.

#### Paratibial and Intratibial injection

1.5 × 10^6^ of Saos‐2‐B‐Luc/mKate2 or HOS‐Luc/mKate2 cells were injected in a total volume of 10 μL Matrigel (Corning, Wiesbaden, Germany) solution at 4 mg/mL, whatever the injection method used. Procedures were performed under a sterile atmosphere and with the mice being anesthetized using 3% isoflurane. Paratibial injection with periosteum denudation and intratibial injection were performed according Uluçkan et al., with some modifications 20.

Paratibial injection was performed applying a 30‐G needle perpendicular to the tibia after a 0.5‐cm skin incision. Before cell injection, periosteum was gently activated with the needle (periosteum denudation).

For intratibial injection, a 0.5‐cm skin incision was performed just below the knee joint and cells were injected into the intramedullary cavity of the tibia with a 30‐G syringe, and then, skin was sutured. To avoid bone pain, an analgesic (buprenorphine at 0.3 mg/kg) was applied in addition to general anesthesia.

Mice were clinically monitored every week, for general symptoms, weight, and tumor size. They were euthanized at the onset of general symptoms (e.g., weight loss, difficulty to walk).

### *In vivo* bioluminescence and CT imaging

Images were acquired using IVIS SpectrumCT (Perkin Elmer). This multimodality imaging system allows the detection of tumors and metastases in X‐ray tomography coregistered with optical images of tagged tumor cells without image adjustment for anatomical correspondence. As light is only emitted by tumor cells without any background signal, bioluminescence is a highly specific and sensitive methodology for tumor detection and follow‐up over time 13. For optical detection, mice were injected intraperitoneally with 150 mg/kg of D‐luciferin (Beetle luciferin, Promega, Charbonnières, France) and then anesthetized with 3% isoflurane. For primary tumor detection, the lower section of the body (area of the lower legs) was imaged. For metastatic spread, especially lung metastases, primary tumor was covered to exclude its signal and chest was imaged. For primary tumors as for metastases, acquisition parameters were automatically computed by the SpectrumCT software to optimize bioluminescence signals (photons per second [p/s]) detection.

### *Ex vivo* organs imaging

After sacrifice, organs (legs, lungs, and spleen) were collected and immersed in 150 μg/mL of D‐luciferin and then imaged individually for luciferase detection using IVIS SpectrumCT system.

### Histology

Organs were fixed in a 4% paraformaldehyde and embedded in paraffin. Tissues were stained with hematoxylin–eosin–saffron (HES) for morphology. Paraffin sections were processed following heat‐induced antigen retrieval using a mouse antifirefly luciferase monoclonal antibody (1:200; ThermoFisher Scientific, Waltham, MA, USA). The cytoplasmic signal was revealed with klear mouse kit (GBI laboratories Washington 98021 USA). Slides were examined using light microscopy (Zeiss, Marly‐Le‐Roy, France). IGR‐N91‐Luc neuroblastoma cells 21 were used as positive control. Single representative whole tumor tissue section from each animal was digitized using a slide scanner NanoZoomer 2.0‐HT (C9600‐13; Hamamatsu Photonics, Massy, France). Histology was reviewed by an expert pathologist of human bone.

### Statistical analysis

*In vitro* and *in vivo* bioluminescence intensity is shown as the mean ± standard error of mean (SEM) using Graphpad Prism^®^ Software version 5.00 (Graphpad Software Inc, La Jolla, CA, USA).

## Results

### Osteosarcoma cell transduction

**Figure 1:**
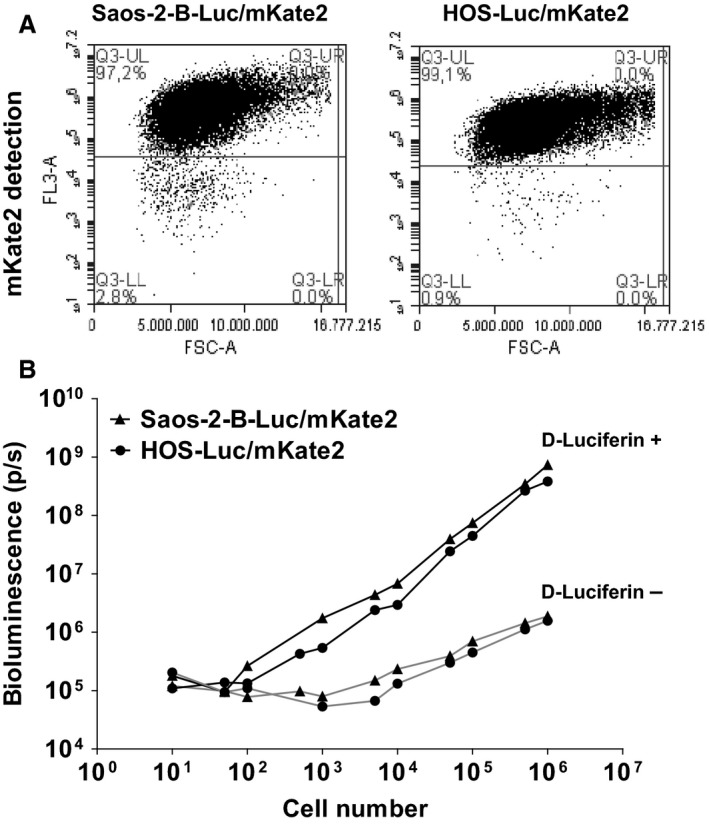
Characterization of luciferase‐transduced osteosarcoma cells. (A) mKate2 (FL3‐A) selection by flow cytometry of transduced Saos‐2‐B‐Luc/mKate2 and HOS‐Luc/mKate2 cells showed a rate of more than 90% positive cells. (B) Bioluminescence detection using IVIS SpectrumCT system showed increased bioluminescence signal paralleling the increase numbers of Plvx‐CAG‐luc‐2A‐mKate2 transfected osteosarcoma cells Saos‐2‐B and HOS (black ▲ and ●, respectively) in the presence of luciferin, but not without luciferin (gray ▲ and ●, for Saos‐2‐B and HOS, respectively).

All seven osteosarcoma cell lines were successfully transduced with a rate above 90% of Luc/mKate2‐positive cells (Fig. 1; Figure S1), including HOS and MG‐63 cell lines after selection by flow cytometry. Data are shown for Saos‐2‐B‐Luc/mkate2 and HOS‐Luc/mKate2 which were also used for the *in vivo* model establishment (Fig. 1). Cell transduction with Plvx‐CAG‐luc‐2A‐mKate2 plasmid using the viral vector resulted in 98% and 68% of luciferase/mKate2‐positive cells for Saos‐2‐B and HOS, respectively. HOS cells were subjected to an additional selection using mKate2 positivity by flow cytometry which resulted in a 99% rate of HOS‐positive cells (Fig. 1A).

Using IVIS system, we were able to detect bioluminescence >10^5^ photons/sec in both Luc/mKate2 transduced cells at a concentration of 1000 cells. Bioluminescence intensity increased with the number of cells in both bioluminescent cell lines in the presence of luciferin substrate (Fig. 1B).

### Tumorigenicity and metastatic potential of osteosarcoma cell lines in an orthotopic setting to the bone using bioluminescence *in vivo*

**Figure 2:**
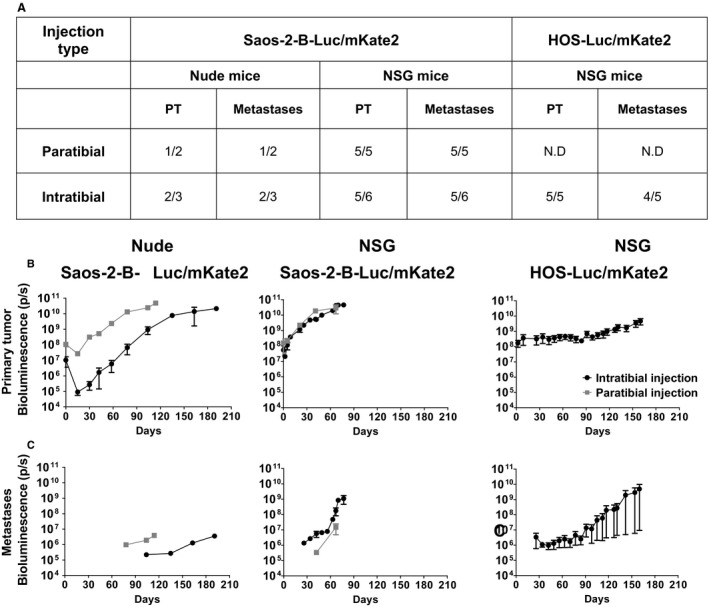
*In vivo* tumor growth and metastatic potential of Saos‐2‐B‐Luc/mKate2‐CDX and HOS‐Luc/mKate2‐CDX orthotopic bioluminescent models. (A) Primary tumor growth engraftment and metastatic rate according to osteosarcoma cell line, mouse strain, and type of injection. (B) Primary tumor *In vivo* bioluminescence detection overtime. (C) Metastases *In vivo* bioluminescence detection overtime. Orthotopic osteosarcoma bioluminescent models: Saos‐2‐B‐Luc/mKate2‐CDX in nude (left panel) and NSG mice (central panel); HOS‐Luc/mKate2‐CDX in NSG mice (right panel). 1.5 × 10^6^ Luc/mKate2 transduced cells were injected in NSG mice by intratibial injection (black) for both cell lines (Saos‐2‐B and HOS). Saos‐2‐B‐Luc/mKate2 was also injected by paratibial injection (gray) on the left tibia for NSG as well as in nude with intratibial and paratibial injection. NSG and nude mice were imaged for bioluminescence with IVIS spectrumCT system until 67 or 77 days (paratibial or intratibial) and 114 or 191 days (paratibial or intratibial), respectively, in Saos‐2‐B‐Luc/mKate2‐CDX and 160 days for NSG in HOS‐Luc/mKate2‐CDX. ND, Not done.

We first developed the Saos‐2‐B‐Luc/mKate2 cell line model. Saos‐2‐B engraftment rate appeared higher (Fig. 2A) and primary tumor (Fig. 2B), and metastases growth (Fig. 2C) was faster in NSG than in nude mice. Bioluminescence was detectable much earlier than clinical deformation of the leg.

Primary tumor bioluminescence was detectable *in vivo* as early as 5 days after Saos‐2‐B‐Luc/mKate2 cell injection (the first evaluation time point) for both mouse strains and both injection conditions used (Fig. 2B). Bioluminescence >10^10^ was reached at 40–50 days and 90–163 days in NSG and nude mice, respectively. Between paratibial and intratibial injection, no difference in primary tumor growth was observed in NSG mice. In nude mice, primary Saos‐2‐B‐Luc/mKate2 tumors showed an initial decrease in bioluminescent signals with a subsequent recovery of tumor growth. This phenomenon was more prominent for intratibial injection (Fig. 2B), resulting in delayed tumor growth. Bioluminescence allowed to detect metastases that occurred earlier in NSG than in nude mice (26–42 and 78–104 days after injection, respectively) (Fig. 2C). In nude mice, metastases occurred earlier after paratibial injection than intratibial injection, as observed for the primary tumors (Fig. 2C). In NSG mice, intratibial injection seemed slightly favorable for metastatic growth as compared to paratibial Saos‐2‐B‐Luc/mKate2‐CDX with first detection at 26 and 42 days, respectively (Fig. 2C).

Consistent with the bioluminescent observations, clinical deformation of the leg appeared later in nude as compared to NSG mice (100 and 40 days, respectively) and later after intratibial injection as compared to paratibial one in NSG mice (60 and 40 days, respectively). Difficulties in moving led to NSG mice sacrifice between 67 and 77 days after paratibial and intratibial injections, respectively, and for nude mice between 114 and 191 days after paratibial and intratibial injections, respectively.

For the HOS‐Luc/mkate2 cell line, we used the best conditions observed with Saos‐2‐B‐Luc/mKate2‐CDX, that is intratibial injection in NSG mice. Primary tumors developed in all five mice injected (Fig. 2A) but barely grew locally (Fig. 2B). Bioluminescence values were 10^7^–10^8^ at day 0 and 3.5 × 10^9^ at day 160 when mice were sacrificed. However, lung metastases were detected 26 days after injection in four of five animals (Fig. 2C). In total, the growth rate of primary tumors and metastases of the intratibial HOS model were slower than those seen with intratibial Saos‐2‐B‐Luc/mKate2‐CDX in NSG. Metastasis bioluminescence values reached >10^8^ at 110 and 70 days, respectively.

### Radiological and morphological characteristics of the orthotopic Saos‐2‐B and HOS osteosarcoma Luc/mKate2‐CDX models

**Figure 3:**
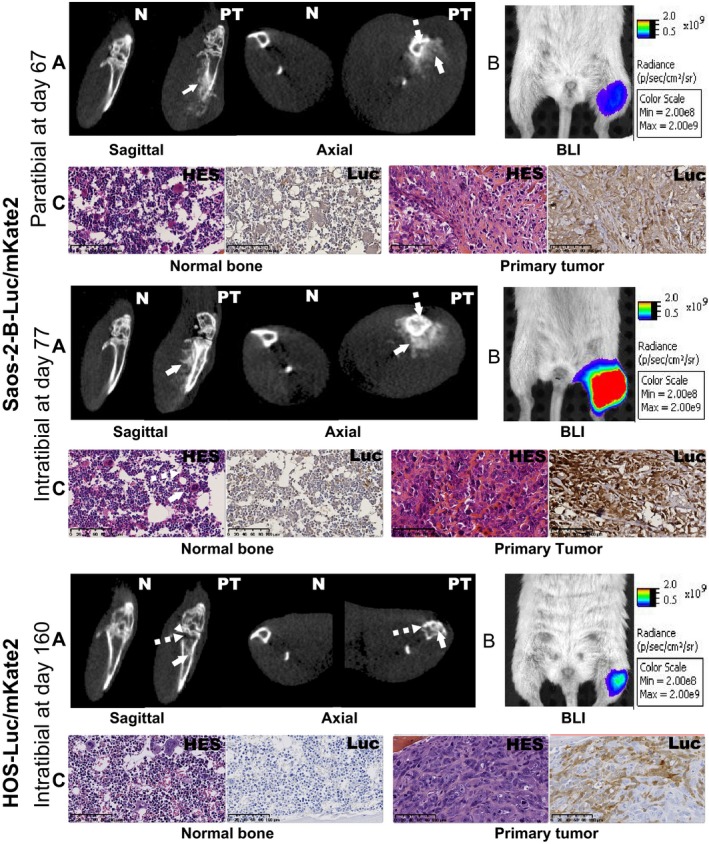
Primary bone tumor—morphological and histological characteristics of SAOS‐2‐B‐Luc/mKate2‐CDX and HOS‐Luc/mKate2‐CDX orthotopic models in NSG mice. Orthotopic osteosarcoma bioluminescent models in NSG mice at sacrifice time: paratibial Saos‐2‐B‐Luc/mKate2‐CDX (top panel), intratibial Saos‐2‐B‐Luc/mKate2‐CDX (middle panel), and intratibial HOS‐Luc/mKate2‐CDX (bottom panel). (A) *In vivo* CT scan imaging by IVIS spectrumCT system of the normal leg (N) and primary tumor (PT), by sagittal and axial view showing osteocondensation (plain arrow) and osteolysis (dotted arrow). (B) *In vivo* bioluminescence imaging by IVIS spectrumCT system of the primary tumor (left leg) compared to the control leg (right leg). (C) Histology using hematoxylin–eosin–saffron (HES) and luciferase stainings of the primary tumor and normal bone at 16× magnification.

CT imaging allowed real‐time detection of tumor growth and modifications of the bone structures in the CDX models (Fig. 3A), but did not detect lung or any other metastases. Saos‐2‐B‐Luc/mKate2‐CDX scans revealed tumor‐bearing tibia bone structure abnormalities similar to those observed in the human disease. Aggressive bone lesions (cortical rupture, periosteal reaction), detection of aberrant new bone formation extending within the extra‐osseous mass (osteocondensation, new calcified material), and some osteolysis (bone destruction) were found as shown in Fig. 3A when mice were sacrificed at day 67 and 77 for paratibial and intratibial, respectively. Osteocondensation was also observed inside the bone of intratibial models, but less in paratibial models (Fig. 3A). These changes were first noted 41 days after Saos‐2‐B‐Luc/mKate2 injection in NSG mice and 78 days in nude mice, independently of injection localization (data not shown). In HOS‐Luc/mKate2‐CDX intratibial model, bone structure alterations had more osteolytic characteristics (Fig. 3A, lower panel) and were detected later (>day 100) with slight osteocondensation only inside the bone detected even later. Overlying the *in vivo* bioluminescence analysis and the CT scan images allowed to confirm that CT abnormalities correspond to the injected human osteosarcoma cells transduced with luciferase in both models (Fig. 3B).

For both Saos‐2‐B‐Luc/mKate2‐CDX and HOS‐Luc/mKate2‐CDX, HES staining confirmed the osteosarcoma nature of primary tumors (osteoid formation), mostly osteoblastic with some fibroblastic components in some animals (Fig. 3C, Table 1). *Ex vivo* bioluminescence analysis (data not shown) and luciferase‐positive staining (Fig. 3C) in bone paraffin‐embedded sections confirmed that histological features correspond to the injected human osteosarcoma cells transduced with luciferase in both models.

**Table 1:** Morphological and histological characteristics of all osteosarcoma bioluminescent orthotopic CDX

Cell line Luc/mKate2	Mouse Strain	Injection Type	Mouse Number	Primary tumor			Metastases					
				Histology		CT	Lung		Bone		Spleen	
				Subtype	Necrosis	Calcification	Histology	BLI	Histology	BLI	Histology	BLI
Saos‐2‐B	Nude	Paratibial	32773	HG OB	−	++	−	++ (78 days)	−	−	−	−
Saos‐2‐B	Nude	Intratibial	33535	HG OB	10%	++	−	++ (100 days)	−	−	−	−
			33536	HG OB	−	+++	−	++ (100 days)	−	+	−	−
Saos‐2‐B	NSG	Paratibial	32752	HG OB	−	+++	−	++	−	N.A	+	+
			32753	HG OB	<1%	+++	−	++	+	N.A	+	+
			32754	HG FB+OB	−	+++	−	++	−	N.A	−	+
			32755	HG FB+OB	−	+++	−	++	−	N.A	+	+
			32756	HG OB	−	+++	−	++	−	N.A	+	+
Saos‐2‐B	NSG	Intratibial	32769	HG OB	−	++++	−	++	−	N.A	+	+
			32770	HG FB+OB	40%	++++	+ (32met)	++++ (Visible)	−	N.A	−	+
			32771	HG OB	−	++++	+ (6met)	++++ (Visible)	−	N.A	+	+
			32772	HG OB	30%	++++	+ (22met)	++++ (Visible)	+	N.A	−	+
			34104	HG OB	−	++++	+ (6met)	+++	+	+	−	+
HOS	NSG	Intratibial	34662	HG FB+OB	−	+	+ (19met)	++	−	−	+	+
			34663	HG FB+OB	−	+	+ (29met)	++	−	+	−	−

BLI, Bioluminescence; CT, computed tomography; FB, fibroblastic subtype; HG, high‐grade osteosarcoma; N.A, not available; OB, osteoblastic subtype; +, positive detection; −, negative detection; Met, metastases.

**Figure 4:**
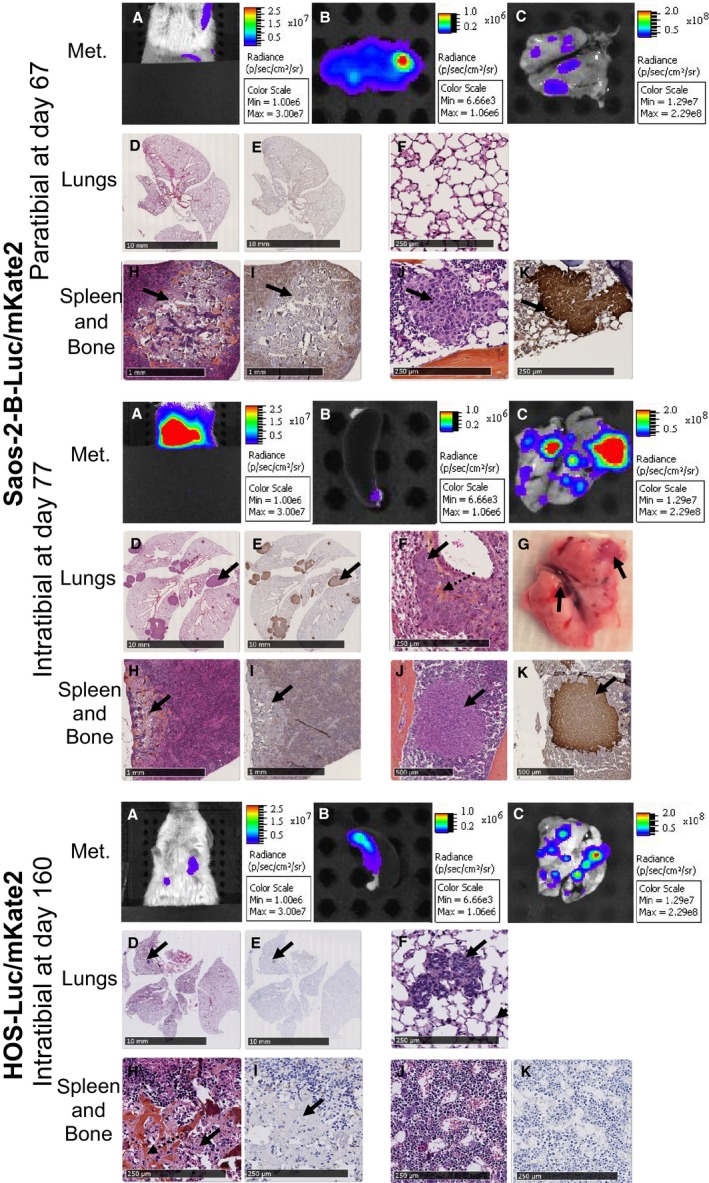
Metastases—morphological and histological characteristics of Saos‐2‐B‐Luc/mKate2‐CDX and HOS‐Luc/mKate2‐CDX orthotopic models in NSG mice. Orthotopic osteosarcoma bioluminescent models in NSG mice at sacrifice time: paratibial Saos‐2‐B‐Luc/mKate2‐CDX (top panel), intratibial Saos‐2‐B‐Luc/mKate2‐CDX (middle panel), and intratibial HOS‐Luc/mKate2‐CDX (bottom panel). *In vivo* bioluminescence of metastases (A). *Ex vivo* bioluminescence of spleen (B) and lungs (C). Lung hematoxylin–eosin–saffron (HES) (D) and luciferase stainings (E) at 0, 24× and 0, 26× magnification for paratibial and intratibial Saos‐2‐B‐Luc/mKate2‐CDX, respectively, and 0, 22× magnification for intratibial HOS‐Luc/mKate2‐CDX. Lung HES staining at 10× magnification (F). Lung macroscopic view (G). Spleen HES (H) and luciferase stainings (I) at 2× magnification (paratibial and intratibial Saos‐2‐B‐Luc/mKate2) and 10× HOS‐Luc/mKate2 intratibial. Bone of not injected leg HES (J) and luciferase stainings (K) at 10× and 4× magnification for paratibial and intratibial, respectively. Plain arrows showed metastases. Dotted arrows showed the intraosseous osteoid matrix. Met, metastases.

*Ex vivo* bioluminescence and histology (HES and luciferase staining) also confirmed the presence of lung metastases in both models (Fig. 4). Saos‐2‐B‐Luc/mKate2‐CDX pulmonary metastases were more frequent and more numerous (range 6–32) when injected intratibially than paratibially in NSG mice as detected by bioluminescence *in vivo* (Fig. 4A) and *ex vivo* (Fig. 4B and C). However, lung metastases in the paratibial model could not be confirmed by histology, despite *in vivo* and *ex vivo* bioluminescent positivity (Fig. 4D–F top panel; Table 1). In intratibial Saos‐2‐B‐Luc/mKate2‐CDX, lung metastases were visible even macroscopically (Fig. 4G). For intratibial HOS‐Luc/mKate2‐CDX, lung metastases were also frequent and numerous (<29) but of smaller size than those in intratibial Saos‐2‐B‐Luc/mKate2‐CDX in NSG mice (Fig. 4D–F; Table 1). Spleen metastases were detected in all model types, except in Saos‐2‐B‐Luc/mKate2‐CDX nude mouse model (Fig. 4H and I). Histology also revealed a unique bone metastasis on the opposite leg (not injected) in two Saos‐2‐B‐Luc/mKate2‐CDX NSG mice (one after intratibial and one after paratibial injections) and one in the homolateral femur of one HOS‐Luc/mKate2‐CDX model detected by *in vivo* and *ex vivo* bioluminescence which could not be detected histologically (Fig. 4J and K).

## Discussion

We developed two novel bioluminescent osteosarcoma orthotopic xenograft models with spontaneous metastatic spread, derived from two osteosarcoma cell lines (Saos‐2‐B and HOS).

We used IVIS SpectrumCT, a multimodality imaging system combining X‐ray tomography (CT scan) with optical detection (bioluminescence), and showed advantages of this technique in our orthotopic bone CDX osteosarcoma models.

The bioluminescence was valuable and presents advantages to detect and follow in real‐time without animal sacrifice, both showed bone primary growth and spread to metastatic sites, especially in the lung. The signal appears before clinical and radiological detection capacity, as previously described 21. We had more difficulties in detecting other metastatic localizations (e.g., bone, spleen) when the *in vivo* bioluminescent signal was close to the background noise, then either *ex vivo* bioluminescent detection or histological confirmation at mice sacrifice was required for metastases detection 21.

CT scans were also valuable and efficient for the analysis of important tumor‐associated bone modifications induced by primary tumor growth, either bone destruction (osteolysis) or aberrant new bone formation (osteocondensation) 14. However, in our models, lung metastases were not detectable by CT scan. IVIS X‐ray capacities are not as good as those reached with a specific X‐ray tomography, giving lower limit detection and resolution. Because of resolution and signal‐to‐noise ratio, tumor volumes under 1 mm remain difficult to detect which could explain the absence of lung metastases detection in CT scans observed in our study.

The combination of different techniques, *in vivo* and *ex vivo* bioluminescence detection, CT scan and histology using HES and luciferase staining allowed us to verify that bone alterations and metastases were due to the presence of the human osteosarcoma cells injected. Thus, these cell lines have the potential to develop primary tumors that mimic different osteosarcoma primary tumors within the *in vivo* bone environment and usual metastases in lung and bone which are the typical metastatic homing observed in patients.

Using the osteosarcoma cell line Saos‐2‐B‐Luc/mKate2, we compared CDX engraftment and metastatic potential within different immune (nude and NSG mice) and bone (intratibial and periosteum‐denuded paratibial injections) contexts. We observed a differential impact of these conditions on the *in vivo* primary bone tumor and metastatic behavior, as reported in other models 22.

The NSG mouse strain used proved to be excellent recipients for osteosarcoma orthotopic xenografts allowing bone tumor engraftment in almost 100% of Saos‐2‐B‐Luc/mKate2‐CDX injected animals in a shorter period of time and more rapid metastatic spread compared to nude mice. The NSG strain also allowed intratibial HOS‐Luc/mKate2‐CDX engraftment in all tested animals and metastatic spread, while the literature reports lack of engraftment in nude mice (subcutaneous and intramuscular injections) 9 and no metastatic potential in SCID mice (paratibial injection) 23. The more profound immune deficiency of NSG mice compared to nude mice (B cell preserved and some innate immunity as macrophages, dendritic cells, and NK cells) not just maximize the chance of xenograft engraftment 19 but favored osteosarcoma primary tumor growth and metastatic spread 14, 21, 24. Lung metastases in Saos‐2‐B‐Luc/mKate2‐CDX models were indeed more frequent in NSG than in nude mice as well as the unusual spleen metastases, not observed in a human context. Spleen metastases were also observed in HOS‐Luc/mKate2‐CDX NSG mice. Others unusual metastatic localizations such as kidney metastases were previously described in 143B‐intratibial CDX Nu/Nu mice models 13, or lymph nodes, liver, adrenal gland, kidney, or ovary in Saos‐2 paratibial CDX in SCID mice 23. These findings suggest the importance of macrophages and innate immunity in osteosarcoma oncogenesis and metastatic potential. Indeed, macrophages intratumor environment is an important aspect of osteosarcoma aggressiveness. High tumor‐associated macrophage (TAM) infiltrates were found associated with better survival and lower risk of metastases 25. Thus, NSG strains might represent an advantage in having osteosarcoma models rapidly developing and spreading to test new drugs. However, therapeutics targeting the immune environment cannot be tested appropriately in these immune deficient mice strains.

Bone is a site composed of many distinct cell types (e.g., osteoblasts, osteoclasts, immune cells) leading to a complex bone microenvironment. This complexity influences the development and progression of osteosarcoma tumors 26, 27. The bone microenvironment allows engraftment and metastatic spread with Saos‐2‐B‐Luc/mKate2‐CDX model in nude mice, while Saos‐2 was described as nontumorigenic after subcutaneous and intramuscular injection in this mouse strain 9. The different bone microenvironment of the primary tumor in Saos‐2‐B‐Luc/mKate2‐CDX model influences primary tumor engraftment and growth behavior as well as metastatic spread. Intratibial models better mimic primary bone tumor, reflecting the range of radiological (CT scan) changes seen in patients with osteosarcoma and developed early, frequent, numerous, and visible lung metastases. In the paratibial setting, lung metastases were not confirmed by histology, although detected by both *in vivo* and *ex vivo* bioluminescence analysis. The metastases might have been missed by the slide sampling, due to their small size. In HOS‐Luc/mKate2‐CDX NSG mouse models, we observed barely any primary bone growth but rapid metastatic spread from day 30, while when injected subcutaneously in NSG mice a fast primary growth within 20 days was described 24, and when injected para‐osseous in SCID do not show metastatic potential 23, highlighting different behaviors in distinct microenvironment context. Recently, tumor microenvironment has been shown to influence drug sensitivity in osteosarcoma MOS‐J syngeneic model using C57BL/6J mice, where a higher response to doxorubicin was observed in intratibial model compared to intramuscular model for tumor growth and necrosis 15.

The genetic background of osteosarcoma may also have influenced *in vivo* behavior in terms of local and metastatic potential. Saos‐2‐B‐Luc/mKate2‐CDX does not express the *TP53 gene* exhibits *RB1* mutation and normal *CDKN2A,* whereas HOS‐Luc/mKate2‐CDX is *TP53* mutated and has *CDKN2A* homozygous deletion 11, 18, hallmarks of aggressive osteosarcoma. When comparing the same *in vivo* conditions (intratibial in NSG mice), the first one has a high local growth potential leading to big osteocondensated aggressive bone tumors, while the second one grew very slowly and is more osteolytic. Lung metastases developed at the same time in both models but grew faster with Saos‐2‐B‐Luc/mKate2‐CDX than in HOS‐Luc/mKate2‐CDX. Genetic transformation of these cell lines (Ki‐RAS transformed HOS cell line, 143B 11, 13 and *in vivo* metastatic selection of Saos‐2 leading to LM7 cell line 23) led to CDX models with higher metastatic potential than the parental cell line: 143B‐subcutaneous CDX models in nude mice presented tumorigenic and metastatic potential while parental HOS was not tumorigenic 11, 13, LM7 paratibial CDX was more metastatic than the parental Saos‐2 in SCID mice 23.

## Conclusion

Our two CDX orthotopic osteosarcoma bioluminescent models with different primary bone behavior and metastatic potential completed those previously published, the “aggressive” HOS‐143B intratibial model in nude mice 13, and the Saos‐2 intrafemoral model in NSG mice 14. These orthotopic models might further help to better follow osteosarcoma human disease in terms of tumor, progression, and metastatic spread, especially under different treatment conditions. They might bring complementary information to other types of existing osteosarcoma models (subcutaneous CDX, syngeneic models in mice or spontaneous osteosarcoma in dogs) 28, with the advantage of real‐time *in vivo* follow‐up in orthotopic and metastatic conditions. Several programs (e.g., MAPPYACTS, IMI2‐P4) are also developing patient‐derived xenograft (PDX) models 29, which are missing in this disease, as well as humanized models. In osteosarcoma, all these multiple models developed in different *in vitro* and *in vivo* contexts are needed to get more insight into the different processes involving osteosarcoma initiation, progression, and treatment sensitivity/resistance.

## Conflict of Interests

The authors declare no potential conflicts of interest.

## Supporting information


**Figure S1.** Characterization of luciferase‐transduced osteosarcoma cells. mKate2 (FL3‐A) selection by flow cytometry of transduced U2OS‐luc/mKate2, 143B‐luc/mKate2, MG‐63‐luc/mKate2, Saos‐2‐luc/mKate2 and IOR/OS18‐luc/mKate2 cells showed a rate of more than 90% positive cells.

## References

[1] Trama, A. , L. Botta , R. Foschi , A. Ferrari , C. Stiller , E. Desandes , et al. 2016 Survival of European adolescents and young adults diagnosed with cancer in 2000–07: population‐based data from EUROCARE‐5. Lancet Oncol. 17:896–906. 27237614 10.1016/S1470-2045(16)00162-5

[2] Bielack, S. S. , S. Smeland , J. S. Whelan , N. Marina , G. Jovic , J. M. Hook , et al. 2015 Methotrexate, doxorubicin, and cisplatin (MAP) plus maintenance pegylated interferon alfa‐2b versus MAP alone in patients with resectable high‐grade osteosarcoma and good histologic response to preoperative MAP: first results of the EURAMOS‐1 good response randomized controlled trial. J. Clin. Oncol. 33:2279–2287. 26033801 10.1200/JCO.2014.60.0734 PMC4486345

[3] Piperno‐Neumann, S. , M.‐C. Le Deley , F. Rédini , H. Pacquement , P. Marec‐Bérard , P. Petit , et al. 2016 Zoledronate in combination with chemotherapy and surgery to treat osteosarcoma (OS2006): a randomised, multicentre, open‐label, phase 3 trial. Lancet Oncol. 17:1070–1080. 27324280 10.1016/S1470-2045(16)30096-1

[4] Ritter, J. , and S. S. Bielack . 2010 Osteosarcoma. Ann. Oncol. 21:vii320–vii325. 20943636 10.1093/annonc/mdq276

[5] Collins, M. , M. Wilhelm , R. Conyers , A. Herschtal , J. Whelan , S. Bielack , et al. 2013 Benefits and adverse events in younger versus older patients receiving neoadjuvant chemotherapy for osteosarcoma: findings from a meta‐analysis. J. Clin. Oncol. 31:2303–2312. 23669227 10.1200/JCO.2012.43.8598

[6] Isakoff, M. S. , S. S. Bielack , P. Meltzer , and R. Gorlick . 2015 Osteosarcoma: current treatment and a collaborative pathway to success. J. Clin. Oncol. 33:3029–3035. 26304877 10.1200/JCO.2014.59.4895 PMC4979196

[7] Omer, N. , M.‐C. Le Deley , S. Piperno‐Neumann , P. Marec‐Berard , A. Italiano , N. Corradini , et al. 2017 Phase‐II trials in osteosarcoma recurrences: a systematic review of past experience. Eur. J. Cancer 75:98–108. 28219023 10.1016/j.ejca.2017.01.005

[8] Martin, J. W. , J. A. Squire , and M. Zielenska . 2012 The genetics of osteosarcoma. Sarcoma 2012:6. 10.1155/2012/627254 PMC3364016 22685381

[9] Mohseny, A. B. , I. Machado , Y. Cai , K. L. Schaefer , M. Serra , P. C. Hogendoorn , et al. 2011 Functional characterization of osteosarcoma cell lines provides representative models to study the human disease. Lab Invest. 91:1195–1205. 21519327 10.1038/labinvest.2011.72

[10] Kunz, P. , J. Fellenberg , L. Moskovszky , Z. Sápi , T. Krenacs , J. Poeschl , et al. 2014 Osteosarcoma microenvironment: whole‐slide imaging and optimized antigen detection overcome major limitations in immunohistochemical quantification. Hartl D, ed. PLoS ONE 9:e90727. 24594971 10.1371/journal.pone.0090727 PMC3940945

[11] Ottaviano, L. , K.‐L. Schaefer , M. Gajewski , W. Huckenbeck , S. Baldus , U. Rogel , et al. 2010 Molecular characterization of commonly used cell lines for bone tumor research: a trans‐European EuroBoNet effort. Genes Chromosom. Cancer 49:40–51. 19787792 10.1002/gcc.20717

[12] Kresse, S. H. , H. Rydbeck , M. Skårn , H. M. Namløs , A. H. Barragan‐Polania , A. M. Cleton‐Jansen , et al. 2012 Integrative analysis reveals relationships of genetic and epigenetic alterations in osteosarcoma. PLoS ONE 7:e48262. 23144859 10.1371/journal.pone.0048262 PMC3492335

[13] Garimella, R. , J. Eskew , P. Bhamidi , G. Vielhauer , Y. Hong , H. C. Anderson , et al. 2013 Biological characterization of preclinical Bioluminescent Osteosarcoma Orthotopic Mouse (BOOM) model: a multi‐modality approach. J. Bone Oncol. 2:11–21. 25688332 10.1016/j.jbo.2012.12.005 PMC4327846

[14] Vormoor, B. , H. K. Knizia , M. A. Batey , G. S. Almeida , I. Wilson , P. Dildey , et al. 2014 Development of a preclinical orthotopic xenograft model of ewing sarcoma and other human malignant bone disease using advanced *in vivo* imaging. Nurminskaya M, ed. PLoS ONE 9:e85128. 24409320 10.1371/journal.pone.0085128 PMC3883696

[15] Crenn, V. , K. Biteau , J. Amiaud , C. Dumars , R. Guiho , L. Vidal , et al. 2017 Bone microenvironment has an influence on the histological response of osteosarcoma to chemotherapy: retrospective analysis and preclinical modeling. Am. J. Cancer Res. 7:2333–2349. 29218254 PMC5714759

[16] Miretti, S. , I. Roato , R. Taulli , C. Ponzetto , M. Cilli , M. Olivero , et al. 2008 A mouse model of pulmonary metastasis from spontaneous osteosarcoma monitored *in vivo* by luciferase imaging. Callaerts P, ed. PLoS ONE 3:e1828. 18350164 10.1371/journal.pone.0001828 PMC2265554

[17] Plessier, A. , L. LeDret , P. Varlet , K. Beccaria , J. Lacombe , S. Mériaux , et al. 2017 New avatars of diffuse intrinsic pontine gliomas (DIPG) from stereotactic biopsies performed at diagnosis. Oncotarget 5:52543–52559. 10.18632/oncotarget.15002 PMC5581049 28881750

[18] Niforou, K. M. , A. K. Anagnostopoulos , K. Vougas , C. Kittas , V. G. Gorgoulis , and G. T. Tsangaris . 2008 The proteome profile of the human osteosarcoma U2OS cell line. Cancer Genomics Proteomics 5:63–78. 18359981

[19] Puchalapalli, M. , X. Zeng , L. Mu , A. Anderson , L. Hix Glickman , M. Zhang , et al. 2016 NSG mice provide a better spontaneous model of breast cancer metastasis than athymic (Nude) mice. Cukierman E, ed. PLoS ONE 11:e0163521. 27662655 10.1371/journal.pone.0163521 PMC5035017

[20] Uluçkan, Z. , A. Segaliny , S. Botter , J. M. Santiago , and A. J. Mutsaers . 2015 Preclinical mouse models of osteosarcoma. Bonekey Rep. 4:670. 25987985 10.1038/bonekey.2015.37 PMC4422092

[21] Daudigeos‐Dubus, E. , L. LE Dret , V. Rouffiac , O. Bawa , I. Leguerney , P. Opolon , et al. 2014 Establishment and characterization of new orthotopic and metastatic neuroblastoma models. In Vivo 28:425–434. 24982206

[22] Quintana, E. , M. Shackleton , M. S. Sabel , D. R. Fullen , T. M. Johnson , and S. J. Morrison . 2008 Efficient tumour formation by single human melanoma cells. Nature 456:593–598. 19052619 10.1038/nature07567 PMC2597380

[23] Ren, L. , A. Mendoza , J. Zhu , J. W. Briggs , C. Halsey , E. S. Hong , et al. 2015 Characterization of the metastatic phenotype of a panel of established osteosarcoma cells. Oncotarget 6:29469–29481. 26320182 10.18632/oncotarget.5177 PMC4745740

[24] Lauvrak, S. U. , E. Munthe , S. H. Kresse , E. W. Stratford , H. M. Namløs , L. A. Meza‐Zepeda , et al. 2013 Functional characterisation of osteosarcoma cell lines and identification of mRNAs and miRNAs associated with aggressive cancer phenotypes. Br. J. Cancer 109:2228–2236. 24064976 10.1038/bjc.2013.549 PMC3798956

[25] Buddingh, E. P. , M. L. Kuijjer , R. A. Duim , H. Bürger , K. Agelopoulos , O. Myklebost , et al. 2011 Tumor‐infiltrating macrophages are associated with metastasis suppression in high‐grade osteosarcoma: a rationale for treatment with macrophage‐activating agents. Clin. Cancer Res. 17:2110–2119. 21372215 10.1158/1078-0432.CCR-10-2047

[26] Alfranca, A. , L. Martinez‐Cruzado , J. Tornin , A. Abarrategi , T. Amaral , E. de Alava , et al. 2015 Bone microenvironment signals in osteosarcoma development. Cell. Mol. Life Sci. 72:3097–3113. 25935149 10.1007/s00018-015-1918-y PMC11113487

[27] Zhang, Y. , Q. Mai , X. Zhang , C. Xie , and Y. Zhang . 2017 Microenvironment signals and mechanisms in the regulation of osteosarcoma *in* Honoki K. and Weiss K. R., eds. Osteosarcoma – biology, behavior and mechanisms. InTechOpen, London, UK.

[28] Mueller, F. , B. Fuchs , and B. Kaser‐hotz . 2007 Comparative biology of human and canine osteosarcoma. Anticancer Res. 27:155–164. 17352227

[29] Stewart, E. , S. M. Federico , X. Chen , A. A. Shelat , C. Bradley , B. Gordon , et al. 2017 Orthotopic patient‐derived xenografts of paediatric solid tumours. Nature 549:96–100. 28854174 10.1038/nature23647 PMC5659286

