# Supplementary figures and images for: Establishment and characterization of in vivo orthotopic bioluminescent xenograft models from human osteosarcoma cell lines in Swiss nude and NSG mice
Source: Cancer Med. 2018 Feb 23;7(3):665–76. doi: 10.1002/cam4.1346 (PMC5852344; doi:10.1002/cam4.1346)

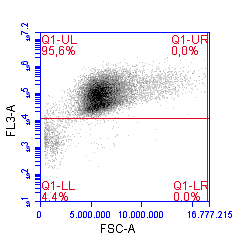


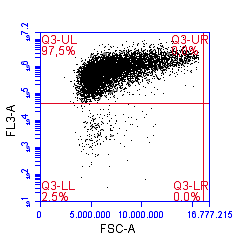


**U2OS-Luc/mKate2 143B-Luc/mKate2**

**HOS IOR/OS 14**

**MG-63-Luc/mKate2 Saos-2-Luc/mKate2**


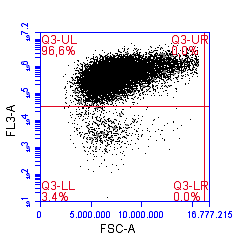

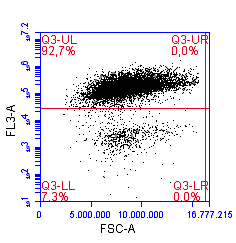


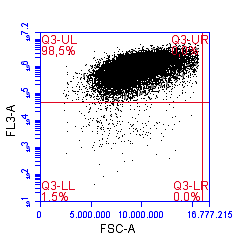


**IOR/OS18-Luc/mKate2**

Supplement: Supplementary file 1 — Figure S1. Characterization of luciferase‐transduced osteosarcoma cells. mKate2 (FL3‐A) selection by flow cytometry of transduced U2OS‐luc/mKate2, 143B‐luc/mKate2, MG‐63‐luc/mKate2, Saos‐2‐luc/mKate2 and IOR/OS18‐luc/mKate2 cells showed a rate of more than 90% positive cells. [file CAM4-7-665-s001.docx]
